# Antihypertensive Mechanism of Orally Administered Acetylcholine in Spontaneously Hypertensive Rats

**DOI:** 10.3390/nu14040905

**Published:** 2022-02-21

**Authors:** Shohei Yamaguchi, Yuzumi Hayasaka, Miho Suzuki, Wenhao Wang, Masahiro Koyama, Yasuko Nagasaka, Kozo Nakamura

**Affiliations:** 1 Department of Science and Technology, Graduate School of Medicine, Science and Technology, Shinshu University, 8304 Minamiminowa, Nagano 399-4598, Japan; 19hs505d@shinshu-u.ac.jp (S.Y.); 20hs502e@shinshu-u.ac.jp (W.W.); 2 Department of Agriculture, Graduate School of Science and Technology, Shinshu University, Nagano 399-4598, Japan; 19as211c@shinshu-u.ac.jp (Y.H.); 19as208c@shinshu-u.ac.jp (M.S.); 3 Wellnas Co., Ltd., Toranomon Masters Building 6F, 1-12-14, Toranomon, Minato-ku, Tokyo 105-0001, Japan; mkoyama32@wellnas.biz; 4 Department of Anesthesia, Tokyo Women’s Medical University Hospital, 8-1 Kawada-cho, Shinjuku-ku, Tokyo 162-0054, Japan; nagasaka.yasuko@twmu.ac.jp; 5 Institute of Agriculture, Academic Assembly, Shinshu University, 8304 Minamiminowa, Nagano 399-4598, Japan

**Keywords:** acetylcholine, hypertension, spontaneously hypertensive rat, sympathetic nervous system, telemetry

## Abstract

Acetylcholine (ACh) acts as a neurotransmitter and neuromodulator. A small dose of eggplant powder rich in ACh (equivalent to 22 g fresh eggplant/d) has been shown to reduce blood pressure (BP) in individuals with higher BP. Here, we investigated the mechanisms underlying the antihypertensive effects of low-dose orally administered ACh in spontaneously hypertensive rats (SHRs). The effects of ACh on BP and sympathetic nervous activity (SNA), including lumbar SNA (LSNA) and renal SNA (RSNA), were evaluated by subjecting conscious SHRs to a telemetry method. Single oral administration of ACh decreased LSNA and lowered BP. Repeated oral administration of ACh for 30 d decreased RSNA and suppressed the elevated BP. Noradrenaline levels in the urine also decreased. However, vagotomy and co-administration of M3 muscarinic ACh receptor antagonist reversed the BP-lowering effect; the dynamics of non-absorbable orally administered ACh was revealed using stable isotope-labeled ACh. In conclusion, ACh acts on the gastrointestinal M3 muscarinic ACh receptor to increase afferent vagal nerve activity, which decreases SNA by autonomic reflex, suppressing noradrenaline release and lowering BP. This study suggests the use of exogenous ACh as an antihypertensive food supplement for controlling the autonomic nervous system, without absorption into the blood.

## 1. Introduction

Acetylcholine (ACh) is an essential endogenous neurotransmitter in several animal taxa [1,2]. During embryonic development, cholinergic nerves develop throughout the body, including the brain, visceral organs, and systemic skeletal muscles. ACh is among the most important endogenous factors that control neural activity in living organisms [3]. The cholinergic system, including ACh receptors and ACh-metabolizing enzymes, is also locally expressed in mammalian tissues (except the nervous system) and even on the inner-body surface, i.e., gastrointestinal mucosa, suggesting that ACh has non-neural and exogenous actions [4,5]. Eggplant-derived ACh (dose: 2.3 mg/d) ameliorated higher blood pressure (BP) in a randomized control trial [6], revealing that exogenous ACh is useful as a functional food substance.

Exogenous ACh obtained from ingredients included in the daily diet may reduce systemic BP. ACh is typically ingested from foods derived from animals with cholinergic systems, but vegetables and fermented foods can also serve as sources of ACh [7]. We recently showed that 19 popular cultivated crops contain ACh, and the content in eggplant was 2900-fold higher than the average ACh content in other crops [8]. Subsequently, it was estimated that the ACh present in 61 g of eggplant is equivalent to the effective dose of 2.3 mg, which can improve BP [9], indicating that ACh derived from eggplant influenced BP following daily ingestion.

We previously evaluated the antihypertensive mechanism of orally administered ACh in spontaneously hypertensive rats (SHRs) using eggplant. Repeated oral administration of eggplant powder containing 10^−8^ mol/kg body weight (BW) of ACh suppressed the increase in BP and catecholamine levels. Eggplant powder relaxed the blood vessels by acting on the M3 muscarinic ACh receptor (M3 mAChR) similar to the mechanism of standard ACh. These results indicate that orally ingested ACh exerts antihypertensive effects by suppressing sympathetic nervous activity (SNA) via M3 mAChR [10]. However, the details of the antihypertensive mechanism of orally administered ACh are unknown. Our latest study revealed a selective BP-lowering effect of orally administered ACh in hypertensive rats, but not in normotensive rats, and indicated the involvement of the suppression of SNA and the dysfunction of the baroreflex [11]. SHRs as an essential hypertension model present excessive SNA [12], as observed in patients with essential hypertension. Therefore, SHRs are suitable for investigating the antihypertensive effects of orally administered ACh on SNA.

SNA is strongly related to the food functionality of exogenous ACh [10,11]. Therefore, SNA must be accurately measured in living tissues. In addition, the levels of catecholamines, which are secreted from the nerve endings and adrenal gland when the sympathetic nervous system is activated, should be investigated to evaluate the actual effect of ACh on BP. During such measurements, rats must be conscious, as the kinetics of nerve activity and catecholamine secretion under anesthesia differ from those under conscious conditions [13]. In the present study, to evaluate the effects of oral ACh on SNA, both renal SNA (RSNA) and lumbar SNA (LSNA), which influence BP, were measured using a telemetry method, which enabled the measurement of SNA and BP in conscious free-moving rats and continuous long-term recording of in vivo data. Urinary catecholamines, adrenaline (AD), and noradrenaline (NAD) were also measured.

Currently, the BP-lowering mechanism of oral ACh, its location of activity, and absorption dynamics are poorly understood, and no information is available on the absorption of orally administered ACh. Typically, functional substances in foods exert their effects after being absorbed into the body. ACh is expected to have low absorbability because of its positive charge and because it is hydrolyzed due to choline esterase in the gastrointestinal mucosa.

In this study, the effects of orally administered ACh on the SNA and BP of conscious SHRs were investigated using telemetry. The long-term effects on RSNA and BP were evaluated following repeated oral administration of ACh. The short-term effects of ACh on SNA, including LSNA and RSNA, were investigated after a single oral administration. In the long- and short-term tests, urinary AD and NAD levels were measured as indicators of SNA. In addition, the action point and nerve pathway of orally administered ACh were investigated using the M3 mAChR antagonist and vagotomy (an operation involving cutting the vagal nerve). The absorption dynamics of orally administered ACh were studied using stable isotope-labeled ACh in the portal vein blood after oral administration.

## 2. Materials and Methods

### 2.1. Chemicals

We used ultrapure water with a specific resistance of 18.2 MΩ cm for the experiments (Ultrapure Water System; Ariumu 611, Sartorius Co., Göttingen, Germany). Methanol (HPLC-grade), formic acid, 5.0 mol/L hydrochloric acid, acetic acid, 2-[4-(2-hydroxyethyl)-1-piperazinyl] ethanesulfonic acid (HEPES), glycerin, and pentobarbital sodium were purchased from Nacalai Tesque (Kyoto, Japan). Acetonitrile (HPLC-grade) and ACh chloride were purchased from Kanto Chemical Co., Inc. (Tokyo, Japan). DL-AD, hydrogen tartrate L-NAD monohydrate, and isoproterenol hydrochloride were purchased from Tokyo Chemical Industry Co., Ltd. (Tokyo, Japan). Isoflurane, perchloric acid, and ketoprofen were purchased from Wako Pure Chemical Industries, Ltd. (Osaka, Japan). Isotonic sodium chloride solution was purchased from Hikari Pharmaceutical Co., Ltd. (Tokyo, Japan). Heparin sodium was purchased from A Y Pharmaceuticals Co., Ltd. (Tokyo, Japan). *N*,*N*,*N*-trimethyl-d9-acetylcholine (d9-ACh) chloride and *N*,*N*,*N*-trimethyl-d9-choline (d9-choline) chloride were purchased from CDN Isotopes Inc. (Quebec, Canada). 1,1-dimethyl-4-diphenylacetoxypiperidinium iodide (4-DAMP) was purchased from Enzo Life Sciences (Farmingdale, NY, USA). Penicillin G potassium was purchased from Meiji Seika Kaisha, Ltd. (Tokyo, Japan). We synthesized (2-aminoethyl) trimethylammonium pivaloylamide (EN) in our laboratory as described previously [8].

### 2.2. Animals and Ethical Statement

SHR/NCrlCrlj were purchased from Charles River Laboratories Japan, Inc. (Kanagawa, Japan). They were kept in plastic cages at Shinshu University (Nagano, Japan) under the following conditions: room temperature, 23 ± 4 °C; humidity, 50 ± 20%; and duration of light and dark cycle, 12 h. Solid chow (MF, Oriental yeast, Tokyo, Japan) and tap water were provided ad libitum. Rats subjected to laparotomy were subcutaneously injected 200,000 U/kg BW of penicillin and 5.0 mg/kg BW of ketoprofen after the operation. The animal experiments and surgical procedures were performed with the approval of the Animal Care Committee of the Faculty of Shinshu University (approval number: 300102). Oral sonde administration to the operated SHRs with telemetry sensors inserted should be avoided, as it may come into contact with and damage the sensor mounting sites, resulting in failed measurements. Voluntary drinking, with water bottles, was adopted instead of a forced administration. The 10^−8^ mol/mL of ACh chloride solution for administration was used at a volume (mL) multiplied with SHR body weight (kg), which was placed in the drinking bottle after dilution to 10^−9^ mol/mL with pure water.

### 2.3. Evaluation of Long-Term Effects of Repeated Oral Administration of ACh on BP and SNA by Telemetry Method

The telemetry system consisted of a telemeter for arterial pressure and SNA measurements (TRM56SP, Millar, Houston, TX, USA), a smart pad receiver (TR180, Millar, Houston, TX, USA), a configurator (TR190, Millar, Houston, TX, USA), a received signal analyzer (PowerLab 16/35, ADInstrument, Sydney, Australia), and waveform analysis software (LabChart Pro v.8.0.5, ADInstrument). The telemeter was placed in 11-week-old male SHRs by making an incision in the abdomen after anesthetization with 3.5% isoflurane, which was reduced to 1.5% for maintenance. The BP sensor was inserted into the abdominal aorta. The telemeter was fixed to the aorta by bioadhesion (Aron Alpha A, Daiichi Sankyo Co., Ltd., Tokyo, Japan). The animals were vivisected on the left ventral side, and the renal sympathetic nervous (RSN) fibers were extracted from the renal artery. A pair of electrodes was placed under the RSN fibers and fixed using a silicone adhesive. The main body of the transmitter was implanted in the abdominal cavity. Finally, the incisions were sutured using a silk thread. After a 1-week recovery period, the operated SHRs were individually listed on a smart pad receiver and assigned to the ACh group (*n =* 6) or the control group (*n =* 6) for the administration of ACh (10^−8^ mol/kg BW) or pure water, respectively; the dose was administered by voluntary drinking from drinking water bottles at 18:00 h (within a few min) for 30 d. The top of the arterial waveform was analyzed as systolic BP (SBP) and bottom as diastolic BP (DBP), and each 24-h average was used as the value of the SBP and DBP for each day. The BP and SNA data measured at 200 Hz were analyzed as follows: RSNA was analyzed to count the spikes of 5 μV and upward for every 2.88 h, and the average number of spikes per day was calculated using the 24-h data. Data from 30 min before and after administration were excluded from the analysis. BP and RSNA are shown as changes from the day before the beginning of the administration.

### 2.4. Evaluation of Long-Term Effects of Repeated Oral Administration of ACh on Catecholamine Excretion and Plasma Angiotensin II (Ang II)

Nine-week-old male SHRs were bred in metabolic cages to collect urine (KN-646, Natsume Seisakusho Co., Ltd., Tokyo, Japan). They were assigned the ACh group (*n* = 6) or control group (*n* = 6), and ACh (10^−8^ mol/kg BW) or pure water, respectively, was orally administered by oral sonde at 18:00 h for 30 d. Urine was collected in a graduated cylinder containing 1 mL of 5.0 mol/L hydrochloride for 24 h after the administration. Urine samples were stored at −80 °C until analysis. After thawing a urine sample, it was centrifuged (5000× *g*, 4 °C, 3 min), and the supernatant was filtered through a syringe filter (pore size: 0.45 µm, Millex-LH, Millipore, Burlington, MA, USA). Urinary catecholamines were quantified using liquid chromatography–tandem mass spectrometry (LC-MS/MS) analysis with the external standard method using an Acquity UPLC system and Quattro Micro API mass spectrometer (Waters Co., Milford, MA, USA) as per the following conditions: column: YMC-Triart PFP (4.6 × 250 mm, 5 µm) and Triart PFP guard column (1.0 × 4.0 mm, 5 µm); mobile phase: 25 mmol/L formic acid−15% (*v*/*v*) methanol-containing water; flow rate: 0.5 mL/min; injection volume: 20 μL; separation temperature: 40 °C; ionization mode: positive electrospray ionization (ESI(+)); analytical mode: multiple reaction monitoring (MRM); capillary voltage: 3.5 kV; source temperature: 120 °C; desolvation temperature: 350 °C; corn gas: 50 L/h; desolvation gas: 600 L/h; MRM transition (*m*/*z*): 166.04 > 56.79 (AD), 152.00 > 106.86 (NAD); cone voltage (V): 25.0 (AD), 25.0 (NAD); collision voltage (eV): 20.0 (AD), 15.0 (NAD). At the end of the experiment, blood was collected from the abdominal aorta of SHRs anesthetized with isoflurane. Blood samples were collected in BD Vacutainer K2 EDTA blood collection tubes (BD Biosciences, Franklin Lakes, NJ, USA) and immediately centrifuged (1500× *g*, 4 °C, 30 min) to obtain plasma samples, which were stored at −80 °C until analysis. LC-MS/MS analyses were conducted at the Research Center for Supports to Advanced Science, Shinshu University. Plasma Ang II concentrations using radioimmunoassay were performed by Aska Pharmaceutical Co., Ltd. (Tokyo, Japan).

### 2.5. Evaluation of Acute Effects of Orally Administered ACh on BP and SNA by Telemetry Method

For this experiment, a telemetry system similar to that used in the long-term study was configured. The telemeter was inserted into 14–16-week-old male SHRs by anesthetizing them with 3.5% isoflurane and then reducing this level to 1.0% with subcutaneous injection of 20 mg/kg BW pentobarbital for maintenance. The BP sensor was inserted into the abdominal aorta through an incision in the femoral artery, and the inserted portion of the catheter was tied and fixed with a suture. In the LSNA measurement group, LSN fibers were separated from the side of the abdominal artery. In the RSNA measurement group, RSN fibers were separated using the procedure described for the long-term study. After an insulating film was laid under the nerves, a bipolar electrode was attached and fixed with a silicone adhesive. The transmitter was placed in the abdominal cavity, and the incisions were sutured. After a 1-day recovery period, the ACh group was administered a single oral dose of ACh (10^−8^ mol/kg BW) from drinking water bottles at 18:00 h; the process took a few min. The control group was given the same amount of pure water using the same method. The BP and SNA data measured at 1000 Hz from 1 h before administration up to 12 h after administration were analyzed as follows. SBP, DBP, RSNA, and LSNA were averaged every 3 h and shown as changes from the average value of 1 h before administration; however, data from 15 min before and after administration were not used in the analysis to exclude the effect of the drinking action.

### 2.6. Evaluation of Acute Effects of Orally Administered ACh on Catecholamine Excretion

Fourteen-week-old male SHRs were bred in metabolic cages (KN-646, Natsume Seisakusho Co., Ltd.). They were assigned to the ACh group (*n* = 6) or the control group (*n* = 6), and ACh (10^−8^ mol/kg BW) or pure water, respectively, was administered orally by oral sonde at 18:00 h. Urine samples accumulated for 24 h were collected using the same method as described in the long-term study and stored at −80 °C until analysis. Catecholamines in urine samples were selectively purified using a spin column (MonoSpin PBA, GL Sciences, Inc., Tokyo, Japan) as per the following procedure: urine samples were thawed at room temperature, and the supernatant was centrifuged (8070× *g*, 3 min, 4 °C). The spin column was centrifuged at 1000× *g* at 4 °C, and 200 μL of 1.0% (*v*/*v*) acetic acid (elution solvent) was added to the solid-phase extraction cartridge. This procedure was repeated once. Next, 200 μL of the urine sample and 380 μL of 1.5 mol/L HEPES-NaOH buffer solution (binding solvent, pH 8.5) and 20 μL of 500 ng/mL isoproterenol solution as a surrogate were added to the column and centrifuged for 2 min. The filtrate was collected, added to the column, and centrifuged for 2 min. We then added 200 μL of 100 mmol/L HEPES-NaOH buffer (wash buffer, pH 8.5) and centrifuged the sample for 1 min; this step was repeated once. Finally, 200 μL of elution solvent was added and centrifuged for 1 min. The eluate was used as an analytical sample. Urinary catecholamines were quantified by LC-MS/MS using the LC-2040c3d and LCMS-8045 systems (Shimadzu Co., Kyoto, Japan) under the following conditions: column: YMC-Triart PFP (4.6 × 250 mm, 5 μm) and Triart PFP guard column (1.0 × 4.0 mm, 5 μm); mobile phase: 25 mmol/L formic acid−15% (*v*/*v*) methanol-containing water; flow rate: 0.50 mL/min; injection volume: 50 μL; separation temperature: 40 °C; ionization mode: ESI(+); analytical mode: MRM; capillary voltage: 4.0 kV; interface temperature: 300 °C; desolvation line temperature: 350 °C; nebulizer gas flow: 3 L/min; heating gas flow: 10 L/min; drying gas flow: 10 L/min; MRM transition (*m*/*z*): 166.05 > 107.15 (AD), 152.00 > 107.10 (NAD), 212.30 > 194.30 (isoproterenol); Q1 pre bias (V): −13.0 (AD), −17.0 (NAD), −20.0 (isoproterenol); collision energy (V): −18.0 (AD), −18.0 (NAD), −10.0 (isoproterenol); Q3 pre bias (V): −24.0 (AD), −21.0 (NAD), −20.0 (isoproterenol).

### 2.7. Investigation of Absorption Dynamics of Orally Administered ACh

Eleven 12–13-week-old male SHRs were subjected to portal vein cannulation. The SHRs were anesthetized with isoflurane at 3.5%, which was reduced to 2.0% for maintenance. After incising the abdomen, the portal vein was exposed and punctured with a 24G needle with clamping. A polyurethane catheter tube (outer diameter: 0.64 mm, internal diameter: 0.30 mm) was inserted into the portal vein and fixed by bioadhesion. The end of the tube was exposed from the back through the subcutis and plugged with a stainless pin. The tube was filled with 50:50 (*v*/*v*) saline/glycerine containing 200 U/mL heparin. After a 1-week recovery period, the catheterized SHRs were starved for 12 h and then orally administered with the stable isotope-labeled ACh (d9-ACh) at a dose of 10^−6^ mol/kg BW (*n* = 5) by oral sonde at 9:00 h. Pure water was administered to the control group (*n* = 6). Portal blood samples (400 μL) each were collected through the catheter before and 0.25, 1, 3, 6, and 9 h after administration. The blood samples were immediately added to 400 μL of 1.6% perchloric acid for enzyme inactivation and deproteinization, followed by centrifugation (1000× *g*, 4 °C, 15 min) to obtain the supernatant. Thereafter, 600 μL of the supernatant, to which internal standard (EN) and 600 μL of 1.0 mol/L phosphate buffer solution (pH 7.0) were added, was added to a weakly acidic cation-exchange solid-phase extraction cartridge (Inertsep CBA 100 mg/mL, GL Sciences Inc., Tokyo, Japan) and washed with pure water. Next, 500 μL of 1.0 mol/L hydrochloric acid solution was added, and the eluate was adjusted to 1.0 mL using the solvent in a volumetric flask. The samples were used to quantify d9-ACh and d9-choline, which is a hydrolyzed product of d9-ACh, by LC-MS/MS using LC-2040c3d and LCMS-8045 systems (Shimadzu Co., Kyoto, Japan). The analysis conditions were as follows: column: YMC-Triart PFP (4.6 × 250 mm, 5 μm); mobile phase: 0.010% (*v*/*v*) formic acid−50% (*v*/*v*) methanol; flow rate: 0.50 mL/min; injection volume: 10 μL; temperature: 40 °C; ionization mode: ESI(+); analytical mode: MRM; MRM transition (*m*/*z*): 155.2 > 69.20 (d9-ACh), 113.2 > 69.20 (d9-choline), 187.3 > 128.15 (EN); Q1 pre bias (V): −11.0 (d9-ACh), −21.0 (d9-choline), −13.0 (EN); collision energy (V): −13.0 (d9-ACh), −22.0 (d9-choline), −14.0 (EN); Q3 pre bias (V): −29.0 (d9-ACh), −27.0 (d9-choline), −24.0 (EN). Each compound was quantified using the standard addition method in accordance with a previous method [8], and the quantitative values were corrected by the recovery of EN.

### 2.8. M3 Muscarinic ACh Receptor Inhibitory Test

We used 4-DAMP, a M3 mAChR selective antagonist, as an inhibitor. Nine-week-old male SHRs were divided into the following groups: ACh (ACh 10^−8^ mol/kg BW; *n* = 6), 4-DAMP (4-DAMP 10^−8^ mol/kg BW; *n* = 6), and ACh + 4-DAMP (ACh 10^−8^ mol/kg BW and 4-DAMP 10^−8^ mol/kg BW). The compounds were dissolved in pure water and administered using oral sonde at 9:00 h. In the ACh + 4-DAMP group, 4-DAMP was administered 10 min before ACh administration. SBP and DBP were measured before and at 3, 6, 9, and 24 h after administration by the tail-cuff method (Softron BP-98A, Softron Co., Tokyo, Japan) as previously reported [14]. BP is shown as a change before and after the administration.

### 2.9. Vagotomy Test

Nine-week-old male SHRs were divided into the vagotomy group (*n* = 3) and the sham group (*n* = 3). The SHRs were anesthetized with isoflurane at 3.5%, which was reduced to 2.0% for maintenance, and were then laparotomized. The vagal nerve fiber at the gastric cardia was exposed and cut in the vagotomy group, and then the incision was sutured. In the control group, a similar process was followed, except for vagotomy. After a 1-week recovery period, ACh (10^−8^ mol/kg BW) was orally administered by oral sonde. SBP and DBP were measured before and 3, 6, 9, and 24 h after administration by the tail-cuff method [14] as described for the receptor inhibition test. BP is shown as the change before and after the administration.

### 2.10. Statistical Analysis

The results are presented as the mean ± standard error (S.E.). The two-tailed Welch’s *t*-test was used to compare the mean values of BP, RSNA, catecholamines, and Ang II in the long-term study, catecholamines in the short-term study, BP in the vagotomy test, and d9-ACh and d9-choline in the oral administration test between the two groups—ACh group and control group. BP, RSNA and catecholamines in the long-term study were analyzed at every data point to evaluate changes in SHRs during the administration period. In the short-term study, the Mann–Whitney *U* test was used to compare the values of BP, RSNA, and LSNA between the two groups. Multiple comparisons of BP among groups for the receptor inhibitory test were performed by Tukey’s test after one-way analysis of variance. *p* < 0.05 was considered to indicate statistically significant results. The two-tailed Welch’s *t*-test was performed using Microsoft Excel 365 MSO (16.0.13901.20276, Microsoft Co., Redmond, WA, USA). Other statistical analyses were performed using SPSS Version 25.0 software (SPSS, Inc., Chicago, IL, USA).

## 3. Results

### 3.1. Chronic Antihypertensive Effect of Repeated Oral Administration of ACh in SHRs

**Figure 1:**
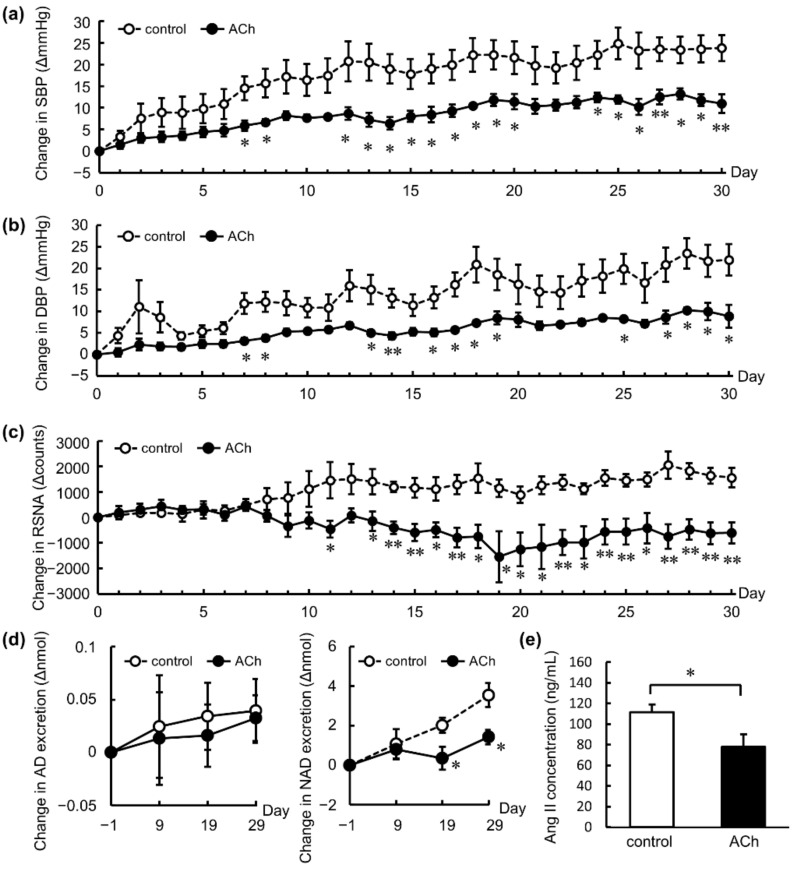
Long-term antihypertensive effects of repeated oral administration of acetylcholine (ACh; dose: 10^−8^ mol/kg body weight) or pure water as a control in spontaneously hypertensive rats. Changes in (**a**) systolic blood pressure (SBP), (**b**) diastolic blood pressure (DBP), and (**c**) count of renal sympathetic nervous activity (RSNA) spikes measured directly using a telemetry method. Changes in excretion of (**d**) urinary catecholamines. The data in (**a**–**d**) were calculated as the change in the values from before administration. Plasma (**e**) angiotensin II (Ang II) concentration on day 30. Each data point and error bar represents mean ± standard error. Data from the long-term study were analyzed by two-tailed Welch’s *t*-test. * *p* < 0.05, ** *p* < 0.01. AD: adrenaline; NAD: noradrenaline.

The SBP and DBP in the control group (received pure water) continuously increased over the test period of 30 d. In contrast, BP elevation was significantly suppressed by orally administered ACh from days 7 to 30 (Figure 1a,b). RSNA was significantly suppressed by oral ACh from days 11 to 30, although it was almost the same between the two groups for the first 7 d (Figure 1c). The urinary excretion of NAD, a major pressor substance released from the adrenal medulla and sympathetic nerve endings, was also significantly suppressed on days 19 and 29 (Figure 1d). Suppression of RSNA and reduction in urinary NAD levels were linked. In addition, orally administered ACh significantly decreased the plasma Ang II levels on day 30 (Figure 1e), indicating that repeated oral administration of ACh chronically lowered vasopressor levels in both the sympathetic nervous system and the renin–angiotensin system of SHRs.

### 3.2. Acute Antihypertensive Effect of Single Oral Administration of ACh in SHRs

**Figure 2:**
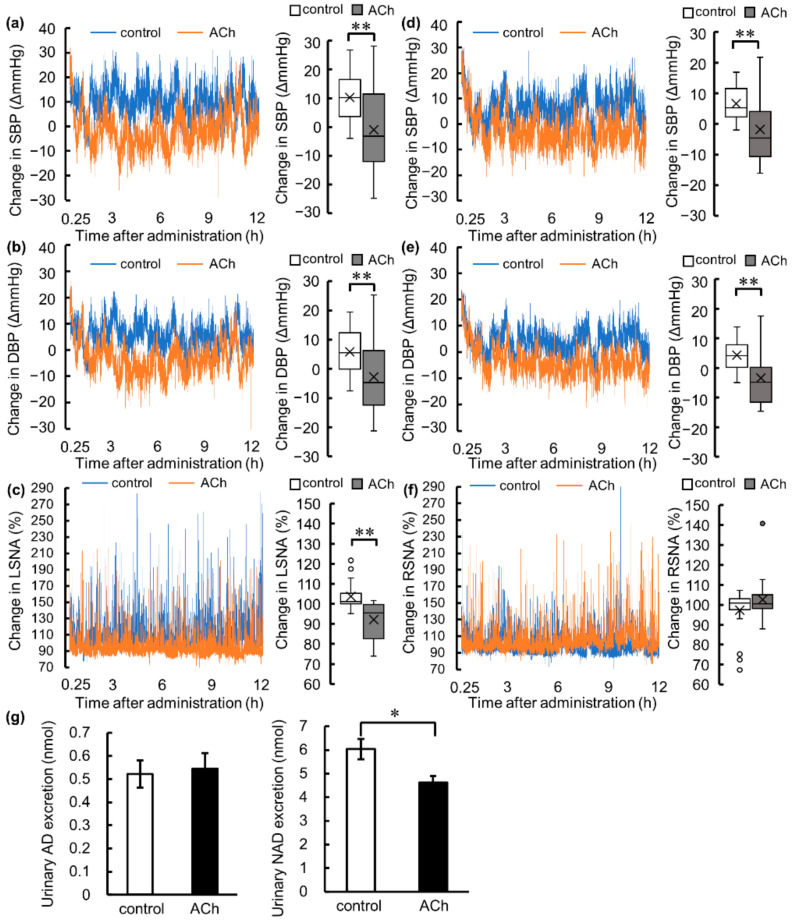
Acute antihypertensive effects of single oral administration of acetylcholine (ACh; dose: 10^−^^8^ mol/kg body weight) or pure water as a control in spontaneously hypertensive rats. Changes in (**a**) systolic blood pressure (SBP), (**b**) diastolic blood pressure (DBP), and (**c**) lumbar sympathetic nervous activity (LSNA) in SHRs in which LSNA was measured using a telemetry method (control [*n* = 6], ACh [*n* = 6]). Changes in (**d**) SBP, (**e**) DBP, and (**f**) renal sympathetic nervous activity (RSNA) in SHRs in which RSNA was measured using the telemetry method (control [*n* = 6], ACh [*n* = 6]). The data in (**a**–**f**) were calculated as change in values from before administration. The time course charts show mean values every 1 s until 12 h after administration and then averaged for each group. The box plots were drawn from values individually averaged every 3 h until 12 h after administration. The data from 15 min after administration were not used in the analysis to exclude the effect of the drinking action. Urinary excretions of (**g**) adrenaline (AD) and noradrenaline (NAD) (control [*n* = 6], ACh [*n* = 6]) are shown; data point and error bar represent the mean ± standard error. The data of telemetry measurement were analyzed using the Mann–Whitney *U* test. The urinal AD and NAD data were analyzed by two-tailed Welch’s *t*-test. * *p* < 0.05, ** *p* < 0.01.

The SBP, DBP, RSNA, and LSNA of conscious free-moving SHRs were measured using the telemetry method from 1 h before and until 12 h after the administration of either ACh or pure water. Administration of oral ACh significantly lowered SBP and DBP concurrently in the same animal and reduction in LSNA was also observed (Figure 2a–c). When measuring RSNA, it was observed that orally administered ACh significantly decreased SBP and DBP; however, it did not affect RSNA (Figure 2d–f). NAD excretion in 24-h urine was significantly lower in the ACh group than in the control group (Figure 2g). Taken together, the reduction of the BP by single oral administration of low-dose ACh could be caused by the reduction in catecholamine release from the suppression of LSNA but not of RSNA.

### 3.3. Action Point and Absorption Dynamics of Orally Administered ACh

**Figure 3:**
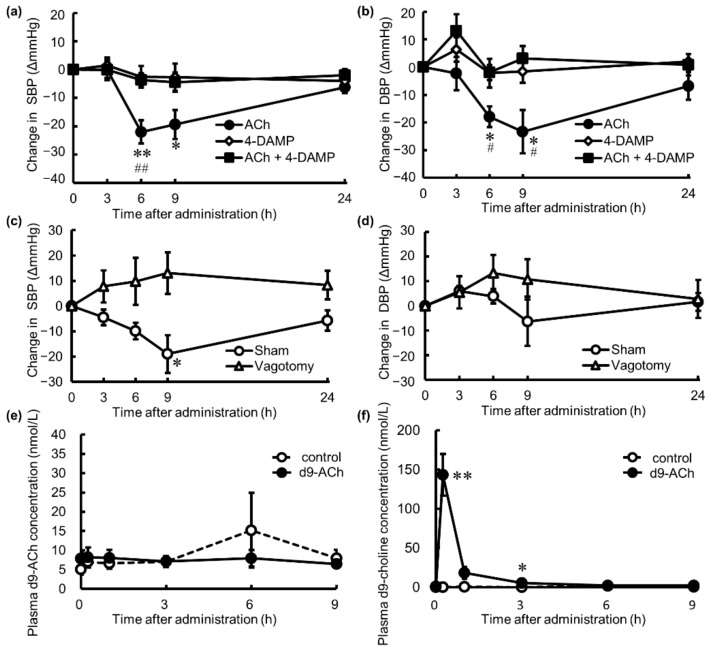
Changes in (**a**) systolic blood pressure (SBP) and (**b**) diastolic blood pressure (DBP) after oral administration of 4-diphenylacetoxy-N-methylpiperidine methiodide (4-DAMP, 10^−8^ mol/kg body weight [BW]), which is an M3 muscarinic acetylcholine receptor (M3 mAChR) antagonist, acetylcholine (ACh, 10^−8^ mol/kg BW), and both substances in spontaneously hypertensive rats (SHRs) (4-DAMP [*n* = 6], ACh [*n* = 6], ACh + 4-DAMP [*n* = 6]). Changes in (**c**) SBP and (**d**) DBP after oral administration of ACh (10^−8^ mol/kg BW) in SHRs with vagotomy or sham operation (vagotomy [*n* = 3], sham [*n* = 3]). BP shown in (**a**–**d**) was measured using the tail-cuff method. Changes in plasma (**e**) stable isotope-labeled ACh (d9-ACh, *N,N,N*-trimethyl-d9-acetylcholine) and (**f**) d9-choline (*N,N,N*-trimethyl-d9-choline) concentrations after oral administration of d9-ACh (10^−6^ mol/kg BW) or pure water as a control (d9-ACh [*n* = 5], control [*n* = 6]). Each data point and error bar represents the mean ± standard error. Data from the receptor inhibitory test were analyzed by one-way analysis of variance, followed by Tukey’s test. * *p* < 0.05, ** *p* < 0.01 versus 4-DAMP group, # *p* < 0.05, ## *p* < 0.01 versus ACh + 4-DAMP group. The data from the vagotomy test and d9-ACh administration test were analyzed by two-tailed Welch’s *t*-test. * *p* < 0.05, ** *p* < 0.01.

To investigate the action point of oral ACh through M3 mAChR and the nerve pathway for the BP-lowering effect, changes in the BP of SHRs were measured after single oral administration of ACh (10^−8^ mol/kg BW) or 4-DAMP (10^−8^ mol/kg BW), which is a selective antagonist of M3 mAChR, or co-administration of ACh and 4-DAMP. Orally administered ACh significantly lowered both SBP and DBP at 6 and 9 h after administration. However, oral 4-DAMP and co-administration of ACh and 4-DAMP did not affect the BP, indicating that 4-DAMP strongly suppressed the BP-lowering effect of ACh (Figure 3a,b). The BP of sham operation rats was lowered by oral ACh but not in vagotomized SHRs, and the change in SBP significantly differed at 9 h after administration (Figure 3c,d). Elimination of the BP-lowering effect of oral ACh showed that binding of ACh to M3 mAChR and nerve transmission of the stimuli via the gastrointestinal vagal nerve were required for the antihypertensive actions. The absorption of oral ACh into the body was investigated by measuring stable isotope-labeled d9-ACh administered orally and its hydrolyzed product d9-choline in portal vein blood; 10^−6^ mol/kg BW of d9-ACh was orally administered to SHRs, and their blood samples were collected through portal vein cannula before and at 0.25, 1, 3, 6, and 9 h after administration. d9-ACh did not increase in the blood collected from the portal vein (Figure 3e), whereas d9-choline was significantly increased for up to 3 h after administration (Figure 3f), indicating that orally administered ACh was not absorbed into the body alone and that choline hydrolyzed in the gastrointestinal tract was absorbed. These results show that low-dose of orally administered ACh induced the antihypertensive effect via the vagal nerve stimuli, i.e., ACh binding to M3 mAChR in the gastrointestinal tract, but not through activities occurring after absorption into the blood.

## 4. Discussion

The antihypertensive compound ACh is ingested daily from various foods and may mildly reduce BP [6,8,9]. In this study, we observed a reduction in BP and SNA following oral administration of ACh in free-moving SHRs. In addition, orally administered ACh was not absorbed into the systemic circulation, but it stimulated the vagal nerve via the M3 mAChR in the gastrointestinal tract. Repeated oral administration chronically suppressed BP elevation along with the suppression of the sympathetic nervous system and the renin–angiotensin system. Single oral administration caused an acute reduction in BP by suppressing the SNA and by lowering catecholamine levels. The suppression of SNA by ACh may occur through autonomic reflexes, in which the afferent parasympathetic nervous input attenuates the efferent sympathetic nervous outflow, i.e., increased activity of the vagal nerves (afferent parasympathetic nerves) suppressed SNA. Hence, the antihypertensive mechanism of orally administered ACh could be as follows: orally administered ACh acts on M3 mAChR in the gastrointestinal tract, thereby, increasing afferent vagal (parasympathetic) nerve activity, causing autonomic reflex-induced suppression of SNA, lowering catecholamine levels, and finally causing acute and chronic BP lowering. The detailed mechanism is discussed below.

Oral Ach regulates the autonomic nervous system and lowers BP. The decisive involvement of afferent vagal nerves, which consist mostly of parasympathetic nerves, was revealed by the elimination of the BP-lowering effect of orally administered Ach in vagotomized SHRs. The parasympathetic and sympathetic nerves are antagonistically regulated by the autonomic reflex. Afferent vagal (parasympathetic) stimulation is transmitted to the rostral ventrolateral medulla, causing a decrease in vasomotor center activity and subsequently suppressing efferent SNA. In SHRs, suppressing the function of the rostral ventrolateral medulla via the caudal ventrolateral medulla impairs response to increased parasympathetic nerve activity, e.g., baroreflex, is impaired, resulting in increased sympathetic outflow; this functional disorder is thought to contribute to the hypertensive status [15]. Therefore, activation of vagal nerves by orally administered Ach may have suppressed LSNA and BP, leading to the counteraction of the impaired function.

To further examine the downstream mechanisms by which Ach reduces BP in hypertensive rats, we measured NAD and AD excretion in freely moving rats. Orally administered Ach acutely decreased LSNA and urinary NAD levels. NAD is released from postganglionic sympathetic nerve endings, acts on the vascular smooth muscular α-AD receptor, and constricts blood vessels, resulting in increased BP. Subsequently, most NAD is reused in the presynaptic terminal, and some is excreted in the urine. Catecholamines in urine can indicate systemic SNA. The LSN innervates wide parts of the hind limbs, is deeply involved in regulating BP in rats, and shows large vascular resistance in the hind limb region [16,17]. Hexamethonium, a ganglion blocker, decreases plasma NAD level and causes BP lowering by reducing hind region vascular resistance in rats [18]. Therefore, a decrease in NAD level due to the suppression of SNA, including LSNA, was predicted as the major cause of acute hypotension caused by Ach. In contrast, RSNA and urinary AD levels did not decrease acutely.

The difference in acute dynamics between LSNA and RSNA following an oral dose of aCh was considered to have been resulted from BP homeostasis by a baroreflex, which is an autonomic reflex that minimizes BP fluctuation. Pressure stimulation of baroreceptors on the carotid sinus and aortic arch increases/attenuates the baroreceptive afferent parasympathetic nerve’s activity and suppresses/enhances efferent SNA via the autonomic reflex to restore BP. The baroreflex of BP lowering by orally administered ACh attenuated the baroreceptive afferent parasympathetic nerves and enhanced the efferent sympathetic nerves, including RSNA and LSNA, to increase BP. However, in SHRs, activation of the sympathetic nervous system is not sufficient to increase the BP because the function of the baroreflex is reduced [19], and thus, the lowered LSNA and BP by orally administered ACh persisted. The baroreflex affects and enhances RSNA more strongly than it affects LSNA [20]; therefore, RSNA did not decrease significantly in our study. Dysfunction of the baroreflex may be responsible for the specific antihypertensive effect of orally administered ACh under only hypertensive conditions. In a previous study, oral ACh administration had no effect on normotensive Wistar-Kyoto rats [11]. Baroreflex dysfunction is also observed in humans with essential hypertension [21]. In our clinical trial of subjects with higher BP, BP lowering using eggplant-derived ACh showed a stronger effect on individuals with grade 1 hypertension than on individuals with normal-high BP [6]. AD is secreted from the adrenal glands through activation of the adrenal sympathetic nerve. The non-decrease in AD caused by oral ACh suggested that the adrenal SNA, which was also strongly affected by the baroreflex as the RSNA [22], was sufficiently enhanced by the baroreflex of BP-lowering via oral ACh.

Upon repeated ACh administration, the RSNA level remained the same during the initial 7 d, indicating that the baroreflex, as well as the single oral administration, activated RSNA. RSNA then began to decrease from day 8 and became significantly low starting on day 11. It was considered that the constantly maintained low BP no longer evoked the baroreflex, leading to significantly decreased RSNA. Urinary NAD level on the day before the test was the same as that on day 9 and was significantly low on days 19 and 29. Urinary AD did not change significantly during the test period. Therefore, repeated oral ACh also attenuated the SNA, including RSNA, and decreased NAD level and suppressed BP elevation. On day 30, plasma Ang II levels were significantly decreased in addition to NAD levels. Ang II, a vasopressor in the renin–angiotensin system involved in chronic hypertension, causes vasoconstriction and promotes aldosterone secretion. Renin, a starting material for the renin–angiotensin system, is secreted from juxtaglomerular cells in the kidney by β1-adrenergic action of catecholamines, indicating that lowered NAD level due to RSNA by orally administered ACh suppressed renin secretion and reduced Ang II level. A reduction in RSNA due to orally administered ACh may also contribute to decreased Ang II level because plasma renin activity was decreased by renal denervation in SHRs in a previous study [23]. It is thought that repeated oral ACh administration suppresses the renin–angiotensin system by decreasing SNA and catecholamine levels. The sympathetic nervous system and the renin–angiotensin system are enhanced in SHRs [12,24]. Ang II promotes NAD release and suppresses NAD reuptake [25]. The crosstalk between the vasopressor systems aggravates hypertension in SHRs. Orally administered ACh suppressed both pressor systems and may have exerted synergistic effects to improve hypertension in SHR.

In the present study, we employed the telemetry method to measure SNAs and BP in conscious and free-moving SHRs. Sensors for measuring these values were inserted under the sympathetic nerve fibers and into the abdominal aorta, and a transmitter was implanted into the body. Directly measured in vivo data were instantly recorded, and the dynamics of RSNA and LSNA after oral administration of ACh under conscious conditions were elucidated. We also demonstrated that orally administered ACh was not absorbed into the body based on the analysis of orally administered d9-ACh in the portal vein blood. Stable isotope-labeled d9-ACh, its molecular weight differs from that of natural ACh, can be used to selectively quantify exogenous ACh from endogenous ACh using mass spectrometry. Blood sampling from individual conscious SHRs can be performed repeatedly using the portal vein cannulation, and the blood sample collected from the portal vein is thought to contain the maximum amount of substance absorbed from the gastrointestinal tract in its original form, as the absorbed substance transfers through the portal vein to the liver for metabolism. The non-absorbed oral ACh could not affect organs inside the body, supporting the finding that afferent vagal nerve innervation of the gastrointestinal tract mediates the antihypertensive effect. In the mammalian gastrointestinal mucosa, in which M3 mAChR is mainly expressed [26], orally administered ACh may act on the M3 mAChR and stimulate the afferent vagal nerve through the release of hormones or neurotransmitters.

Gamma amino butyric acid (GABA), another antihypertensive compound in food, is thought to suppress SNA and NAD release by acting on GABA_B_ receptors expressed on peripheral nerve cells after absorption into the body [27,28]. However, no data on SNA have been reported yet. The effective dose for the chronic effect of orally administered ACh in the present study was 1/291 of the lowest effective dose of GABA on SHRs (0.00291 mmol/kg BW) [27]. The lower effective dose of orally administered ACh is attributed to the hypotensive effects on the double major pressor systems, i.e., the sympathetic nervous system and the renin–angiotensin system. Alternatively, orally administered ACh acts on the gastrointestinal tract via the vagal nerve, which has the largest distribution area among the cranial nerves. Orally administered ACh is not absorbed as ACh, instead; it is absorbed as choline, an essential and safe nutrient approved in the United States, indicating the safety of orally administered ACh in metabolism. The increase in blood choline level in the present study following the oral ACh was too low to affect the muscarinic receptors [29] and would have no effect on BP and SNA. ACh is widespread in nature and is found in a wide variety of foods [8]. Non-absorbable orally administered ACh from foods evaluated in this study is safe for consumption. Therefore, we do not have to worry about the side effects of absorbed ACh in vivo, such as excessive hypotension and bradycardia. In this study, the absorption dynamics data of oral ACh indicate that ACh is safe as a functional food component.

## 5. Conclusions

Repeated oral administration of ACh showed antihypertensive effects by suppressing the sympathetic nervous and renin–angiotensin systems. Direct measurement of SNA in conscious free-moving SHRs using the telemetry method revealed the suppression of SNAs as an antihypertensive mechanism of orally administered ACh. ACh, which functions through the afferent vagal nerve in the gastrointestinal tract without absorption, is a promising and safe antihypertensive food component for improving and preventing hypertension. Our study provides insight into using ACh as a functional food factor.

## Data Availability

The data that support the findings of this study are available from the corresponding author, K.N., upon reasonable request.
